# Supplementary material for: The Systems Biology Research Tool: evolvable open-source software
Source: BMC Syst Biol. 2008 Jun 29;2:55. doi: 10.1186/1752-0509-2-55 (PMC2446383; doi:10.1186/1752-0509-2-55)
Supplement: Additional file 1 — SBRT Archive. An archive of the current version of the Systems Biology Research Tool. [file 1752-0509-2-55-S1.zip › sbrt-1.4.0/doc/developers_guide/api/sbrt/kernel/fba/CatalyzedFluxome.html]

CatalyzedFluxome


|  |  |  |
| --- | --- | --- |
| **Overview** \| **Package** \| **Class** \| **Use** \| **Tree** \| **Deprecated** \| **Index** \| **Help** | |  |
| **PREV CLASS**   **NEXT CLASS** | **FRAMES**    **NO FRAMES**     **All Classes** |  |
| SUMMARY: NESTED \| FIELD \| CONSTR \| METHOD | DETAIL: FIELD \| CONSTR \| METHOD |  |


---


## sbrt.kernel.fba Interface CatalyzedFluxome

**All Superinterfaces:**: Fluxome

**All Known Subinterfaces:**: IrrevRxnFluxome

**All Known Implementing Classes:**: IrrevRxnFluxomeV1

---

``` public interface CatalyzedFluxome extends Fluxome ```

This interface is used to represent fluxomes with catalyzed reactions.

**Author:**
:   This interface was written and documented by Jeremiah Wright
    while in the Wagner lab.

---

| **Method Summary** | |
| --- | --- |
| `boolean` | `areReqCatalysts(java.util.Collection<java.lang.String> catalysts, java.lang.String rxnName)`             Indicates if the provided collection of catalysts is required for the reaction with the specified name. |
| `int` | `catalysts()`             Returns the total number of catalysts contained in this fluxome. |
| `BooleanRelation` | `getCatalystRule(java.lang.String rxnName)`             Returns the boolean logic rule for catalysts of the reaction with the provided name. |
| `java.util.Map<java.lang.String,? extends BooleanRelation>` | `getCatalystRules()`             Returns the boolean logic rule for the catalysts of all reactions in this fluxome. |
| `java.util.Set<java.lang.String>` | `getCatalysts()`             Returns the set of all catalysts contained in this fluxome. |
| `java.util.Set<java.lang.String>` | `getRxnNames(java.lang.String catalyst)`             Returns the set of names of all chemical reactions contained in this fluxome that are catalyzed by the provided catalyst. |
| `java.util.Map<java.lang.String,? extends ChemRxn>` | `getRxns(java.lang.String catalyst)`             Returns the chemical reactions contained in this fluxome that are catalyzed by the provided catalyst. |
| `boolean` | `isCatalyst(java.lang.String s)`             Indicates if the provided string is the name of a catalyst contained in this fluxome. |
| `boolean` | `isReqCatalyst(java.lang.String catalyst, java.lang.String rxnName)`             Indicates if the provided catalyst is required for the reaction with the provided name. |
| `CatalyzedFluxome` | `replaceConstraints(FluxConstraints constraints, double constraintTolerance)`             Returns a copy of this fluxome, but with all constraints replaced by those provided. |

**Methods inherited from interface sbrt.kernel.fba.Fluxome**

`addedConstraints, areRxnNames, exchangeRxns, getConstraints, getEquivRxnNames, getEquivRxnNames, getExchangeRxnNames, getExchangeRxns, getFluxConstraint, getInternalRxnNames, getInternalRxns, getReverseRxnNames, getRxn, getRxnExprConstraint, getRxnNames, getRxns, getRxns, getSpecies, getStoichiometryMatrix, internalRxns, isFluxVector, isRxnName, isSpecies, isValid, redundantRxns, rxns, species`

**Method Detail**

### getRxns

```
java.util.Map<java.lang.String,? extends ChemRxn> getRxns(java.lang.String catalyst)
```

:   Returns the chemical reactions contained in this fluxome
    that are catalyzed by the provided catalyst.

    :   **Parameters:**: `catalyst` - the name of the catalyst whose reactions are to be returned. **Returns:**: reaction names mapped to their respective chemical reactions.

---


### getRxnNames

```
java.util.Set<java.lang.String> getRxnNames(java.lang.String catalyst)
```

:   Returns the set of names of all chemical reactions
    contained in this fluxome that are catalyzed by the provided
    catalyst.

    :   **Parameters:**: `catalyst` - the name of the catalyst whose reaction names are to be returned. **Returns:**: the set of names of all chemical reactions contained in this fluxome that are catalyzed by the provided catalyst.

---


### getCatalystRule

```
BooleanRelation getCatalystRule(java.lang.String rxnName)
```

:   Returns the boolean logic rule for catalysts of the reaction with the
    provided name.

    :   **Parameters:**: `rxnName` - the name of the reaction whose catalyst rule are to be returned. **Returns:**: the boolean relationship of the catalysts of the specified reaction.

---


### getCatalystRules

```
java.util.Map<java.lang.String,? extends BooleanRelation> getCatalystRules()
```

:   Returns the boolean logic rule for the catalysts of all reactions in
    this fluxome.

    :   **Returns:**: reaction names mapped to the boolean relationship of their catalysts.

---


### getCatalysts

```
java.util.Set<java.lang.String> getCatalysts()
```

:   Returns the set of all catalysts contained in this fluxome.

    :   **Returns:**: the set of all catalysts contained in this fluxome.

---


### isCatalyst

```
boolean isCatalyst(java.lang.String s)
```

:   Indicates if the provided string is the name of a catalyst
    contained in this fluxome.

    :   **Parameters:**: `s` - the string to test. **Returns:**: `true` if the string is a valid catalyst; `false` otherwise.

---


### catalysts

```
int catalysts()
```

:   Returns the total number of catalysts contained in this fluxome.

    :   **Returns:**: the total number of catalysts contained in this fluxome.

---


### isReqCatalyst

```
boolean isReqCatalyst(java.lang.String catalyst,
                      java.lang.String rxnName)
```

:   Indicates if the provided catalyst is required for the reaction with
    the provided name.

    :   **Parameters:**: `catalyst` - the catalyst.: `rxnName` - the name of the reaction. **Returns:**: `true` if the provided catalyst is required by the reaction with the provided name; `false` otherwise.

---


### areReqCatalysts

```
boolean areReqCatalysts(java.util.Collection<java.lang.String> catalysts,
                        java.lang.String rxnName)
```

:   Indicates if the provided collection of catalysts is required for the
    reaction with the specified name.

    :   **Parameters:**: `catalysts` - the collection of catalysts.: `rxnName` - the name of the reaction. **Returns:**: `true` if the provided catalysts are required by the reaction with the provided name; `false` otherwise. **Throws:**: `java.lang.IllegalArgumentException` - if `rxnName` is not a valid reaction name.

---


### replaceConstraints

```
CatalyzedFluxome replaceConstraints(FluxConstraints constraints,
                                    double constraintTolerance)
```

:   Returns a copy of this fluxome, but with all constraints
    replaced by those provided. If a flux constraint for a
    particular reaction was not provided, its default value
    will be used.

    :   **Specified by:**: `replaceConstraints` in interface `Fluxome`
    :   **Parameters:**: `constraints` - the new set of flux constraints on the system.: `constraintTolerance` - the allowed amount by which constraints can violate their maximum defined intervals. These maximum defined intervals are implementation-dependent. **Returns:**: a copy of this fluxome, but with all constraints replaced by those provided.


---


|  |  |  |
| --- | --- | --- |
| **Overview** \| **Package** \| **Class** \| **Use** \| **Tree** \| **Deprecated** \| **Index** \| **Help** | |  |
| **PREV CLASS**   **NEXT CLASS** | **FRAMES**    **NO FRAMES**     **All Classes** |  |
| SUMMARY: NESTED \| FIELD \| CONSTR \| METHOD | DETAIL: FIELD \| CONSTR \| METHOD |  |


---
